# Genome-wide CRISPR/Cas9 screen reveals JunB downmodulation of HIV co-receptor CXCR4

**DOI:** 10.3389/fmicb.2024.1342444

**Published:** 2024-05-20

**Authors:** William J. Schulze, Devon A. Gregory, Marc C. Johnson, Margaret J. Lange

**Affiliations:** Department of Molecular Microbiology and Immunology, University of Missouri, Columbia, MO, United States

**Keywords:** JunB, CXCR4, CRISPR/Cas9, HIV, host-factors

## Abstract

HIV-1 relies extensively on host cell machinery for replication. Identification and characterization of these host-virus interactions is vital to our understanding of viral replication and the consequences of infection in cells. Several prior screens have identified host factors important for HIV replication but with limited replication of findings, likely due to differences in experimental design and conditions. Thus, unidentified factors likely exist. To identify novel host factors required for HIV-1 infection, we performed a genome-wide CRISPR/Cas9 screen using HIV-induced cell death as a partitioning method. We created a gene knockout library in TZM-GFP reporter cells using GeCKOv2, which targets 19,050 genes, and infected the library with a lethal dose of HIV-1_NL4-3_. We hypothesized that cells with a knockout of a gene critical for HIV infection would survive while cells with a knockout of a non-consequential gene would undergo HIV-induced death and be lost from the population. Surviving cells were analyzed by high throughput sequencing of the integrated CRISPR/Cas9 cassette to identify the gene knockout. Of the gene targets, an overwhelming majority of the surviving cells harbored the guide sequence for the AP-1 transcription factor family protein, JunB. Upon the generation of a clonal JunB knockout cell line, we found that HIV-1_NL4-3_ infection was blocked in the absence of JunB. The phenotype resulted from downregulation of CXCR4, as infection levels were recovered by reintroduction of CXCR4 in JunB KO cells. Thus, JunB downmodulates CXCR4 expression in TZM-GFP cells, reducing CXCR4-tropic HIV infection.

## 1 Introduction

Human immunodeficiency virus (HIV) remains a significant challenge worldwide despite the success of antiretroviral therapy. As most antiretroviral therapies target viral proteins, promoting viral evolution, HIV resistance to these therapies is highly problematic. HIV relies extensively upon host cell machinery for replication. Thus, targeting host factors required for HIV replication may be therapeutically advantageous to decrease the likelihood of viral evolution resulting in the emergence of drug resistance.

There are over 60 well-characterized host factors reported to impact HIV replication, targeting a variety of different replication stages (Friedrich et al., 2011). For example, cluster of differentiation 4 (CD4), C-C chemokine receptor type 5 (CCR5), and C-X-C motif chemokine receptor 4 (CXCR4) are the primary receptor and co-receptors required for HIV fusion and entry (Mizukami et al., 1988; Bazan et al., 1998). Viruses can use a specific co-receptor or a combination of co-receptors, defining their tropism (R5-, X4-, or dual-tropic) (Berger et al., 1998). R5-tropic HIV is known to be less pathogenic but represents an early stage of infection. In addition, R5 virus is more often associated with transmission. Conversely, X4-tropic HIV is characterized by cell-to-cell fusion (syncytia), a hallmark of disease progression toward acquired immunodeficiency syndrome (AIDS) (Schuitemaker et al., 1992; Connor et al., 1997). CCR5 has been exploited for therapeutic use in multiple ways. The CCR5 inhibitor, Maraviroc (Fätkenheuer et al., 2005), retains its antiretroviral effects in populations with drug resistance to other virus-targeting therapeutics (Gulick et al., 2008). In addition to small molecule inhibitors, multiple patients have been confirmed to be cured of HIV after receiving stem cell transplantation from donors homozygous for a 32 base-pair deletion in the CCR5 gene (Hütter et al., 2009; Gupta et al., 2019; Hsu et al., 2023; Jensen et al., 2023). The success in stem cell transplants has inspired work to edit hematopoietic stem cells using CRISPR for transplantation to improve the probability for haplotype matching (Xu et al., 2019). These examples demonstrate the potential utility of targeting host factors; however, CCR5-based therapies are effective only for R5-tropic HIV. Notably, cells encoding the homozygous deletion in CCR5 can remain susceptible to X4-tropic HIV infection (Michael et al., 1998). Host factors involved in viral replication processes other than viral attachment and fusion have also been identified and investigated for therapeutic development. For example, lens epithelium-derived growth factor (LEDGF), a host factor that facilitates viral integration (Maertens et al., 2004; Vanegas et al., 2005), may also be a promising target, especially in combination with FDA-approved integrase inhibitors (Christ et al., 2010). As it is likely that other therapeutically targetable host factors exist, continued identification and investigation of host factors is a promising endeavor in the search for a functional cure.

The development of siRNA, shRNA, and CRISPR/Cas9 guide RNA (gRNA) libraries has enabled implementation of high throughput screens for host factors involved with HIV infection and latency (Kok et al., 2009; Börner et al., 2010; Park et al., 2017; OhAinle et al., 2018; Dai et al., 2022). Nearly 1,000 potential host factor genes have been identified across various screens. However, as each screen utilized different libraries, partition methods, cell types, and HIV strains, very few of the identified genes overlapped among screens. Furthermore, the role of most of the identified genes in HIV infection remains unclear.

We sought to corroborate previous findings or identify novel host factors required for HIV-1 infection using HIV-1_NL4-3_ mediated cell death as a partitioning method. We generated library of CRISPR/Cas9-edited TZM-GFP cells using the GeCKOv2 pooled CRISPR library developed by the Zhang laboratory at MIT (Sanjana et al., 2014) and infected them with a lethal dose of X4-tropic HIV-1_NL4-3_. We hypothesized that cells with knockout of a gene required for productive infection would survive, while cells with knockouts in genes not required for infection would undergo cell death and be removed from the population. Sequencing of the surviving cell population led to the identification of a previously unidentified host factor, JunB, as necessary for HIV-1 infection. JunB is a member of the AP-1 transcription factor family. The Jun subfamily within the AP-1 family includes c-Jun, JunD, and JunB. JunB can homodimerize or heterodimerize with other AP-1 family members to drive differential transcriptional profiles that promote diverse cellular processes, including proliferation and death (Chiu et al., 1989; Shaulian and Karin, 2002; Garces de los Fayos Alonso et al., 2018). This promiscuity presents a challenge in resolving individual contributions to various regulatory processes and many of the elucidated roles cell type specific. Of note, AP-1 family members including JunB, as well as numerous other transcription factors, have long been implicated in transcription of the HIV provirus (Kaczmarek et al., 2013), as they can bind to palindromic regions in the HIV long terminal repeat (Canonne-Hergaux et al., 1995) to drive HIV transcription (Roebuck et al., 1996). Here, we find that knockout of JunB downmodulates CXCR4 expression in TZM-GFP cells, blocking HIV-1_NL4-3_ infection of these cells. However, whether the downmodulation is due to direct or indirect mechanisms remains unclear. To our knowledge, JunB-mediated downmodulation of CXCR4 has not been previously described.

## 2 Materials and methods

### 2.1 Reagents

Unless otherwise noted, all chemicals were purchased from Sigma-Aldrich (St. Louis, MO). Restriction enzymes and T4 DNA ligase used for cloning purposes were purchased from either New England Biolabs (Ipswitch, MA) or ThermoFisher Scientific (Waltham, MA). Primers were purchased from Integrated DNA Technologies (Coralville, IA).

### 2.2 Plasmids

The Human GeCKOv2 CRISPR knockout pooled libraries 1 and 2 (Addgene # 1000000048) and LentiCRISPRv2 (Addgene #52961) were kind gifts from Dr. Feng Zhang (Sanjana et al., 2014). The packaging vector, psPAX2, was a kind gift from Dr. Didier Trono (Addgene # 12260). The vesicular stomatitis virus glycoprotein (VSV-G) expression plasmid for viral pseudotyping, pMD-G, was obtained from Invitrogen (Carlsbad, CA). The plasmid for expression of CXCR4, pcDNA3.1-CXCR4, was a gift from Dr. Erik Procko (Addgene #98942). The plasmid for generation of replication competent HIV-1_NL4-3_ (pNL4-3) was obtained through the NIH AIDS Reagent Program courtesy of Dr. Malcom Martin (ARP-114). The plasmid for expression of the CCR5-tropic HIV-1 envelope glycoprotein (p96ZM651gp160-opt) was obtained from the NIH AIDS Reagent Program, courtesy of Drs. Yingying Li, Feng Gao, and Beatrice H. Hanh (ARP-8662). An HIV-1_NL4-3_-derived proviral plasmid deleted for vif, vpr, vpu, nef and env and encoding a CMV-driven enhanced green fluorescent protein (pNL4-3.gag-pol) was kindly provided by Vineet KewalRamani (National Cancer Institute, Fredrick, MD) for use in single cycle infectivity assays. A second proviral vector (pNL4-3.gag-env) derived from pNL4-3.gag-pol was engineered to remove pol and restore env in place of CMV-EGFP, allowing generation of viral particles displaying X4-tropic Env and expression of X4-tropic Env upon transduction. LentiCRISPRv2-JunB was constructed using the Zhang lab protocol for CRISPR cloning (Shalem et al., 2014; Dai et al., 2022) using the guide sequence for JunB (see Supplementary Table S1).

### 2.3 Cell lines and virus production

The TZM-GFP cells (TZM WT), Hela-derived GFP reporter cells engineered to overexpress CD4 and CCR5 and have endogenous CXCR4 expression, were a generous gift from Dr. Massimo Pizzato (Rosa et al., 2015). The human cell lines, HEK293FT (Invitrogen, Carlsbad, CA) and TZM-GFP (Rosa et al., 2015), and the CRISPR/Cas9-modified cell line, TZM-GFP-JunB-KO (further described below) were maintained in standard cell culture medium containing Dulbelcco’s Modified Eagle Medium (DMEM, Corning, Corning, NY) supplemented with 7.5% fetal bovine serum (Gibco, ThermoFisher Scientific, Waltham, MA) and 2 mM L-glutamine (Sigma-Aldrich, St. Louis, MO). Cells were incubated at 37°C with 5% carbon dioxide and split at least twice per week using TryplExpress (Gibco, ThermoFisher Scientific, Waltham, MA).

Viruses were produced by transfection of HEK293FT cells. Lentiviral particles encoding the GeCKOv2 pooled CRISPR library or the JunB-specific gRNA were generated as previously described (Shalem et al., 2014; Dai et al., 2022). Replication competent HIV-1_NL4-3_ was generated by transfection of 10 μg pNL4-3 with polyethylenimine (PEI) in 10 cm dishes. X4-tropic single-cycle virus was generated via co-transfection of 500 ng pNL4-3.gag-env, and 500 ng psPAX2 with PEI in 6-well plates. R5-tropic virus was generated by co-transfection of 900 ng pNL4-3.gag-pol and 100 ng p96ZM651gp160-opt with PEI in 6-well plates. VSV-G-pseudotyped virus was generated by co-transfection of 450 ng pNL4-3.gag-env, 450 ng psPAX2 and 100 ng pMD-G in 6-well plates. Virus-containing supernatant was harvested 48 h post-transfection, centrifuged at 1000 × *g* for 5 min to remove cellular debris, and transferred to a new tube. LentiCRISPRv2 viruses, which do not induce GFP expression in TZM-GFP cells, were titered qualitatively by transduction of TZM WT cells, followed by puromycin treatment and cell counting 24-h post puromycin treatment. The approximate multiplicity of infection (MOI) utilized was 0.1 viruses per cell, based on our initial post-selection cell counting results. All other viruses were titered on TZM WT cells via detection of virus induced GFP using a BD Accuri Flow Cytometer (BD BiosciencesFranklin Lake, NJ) or an Attune NxT Flow Cytometer (ThermoFisher Scientific, Waltham, MA). Viruses were stored at −80°C.

### 2.4 Genome-wide CRISPR screen

TZM WT cells were transduced with the CRISPR/Cas9 library generated above at a low MOI, qualitatively determined as described in section 2.3, to reduce the potential for multiple transduction events. Notably, it is possible that multiple transduction events can also occur at low MOI. After 48 h, cells were trypsinized and transferred to a new dish. Puromycin was then added to the culture medium at a concentration of 1 μg/mL to select for transduced cells, generating the starting GeCKOv2 library. The cells were allowed to proliferate over one week and representative aliquots were frozen down in liquid nitrogen. As the GeCKOv2 library contains six guide sequences each for 19,050 genes and control guide sequences, approximately 125,000 cells would represent full library coverage. To qualitatively examine the diversity of the starting GeCKOv2 library, genomic DNA was isolated from a portion of the starting library cells using the DNeasy Blood and Tissue kit (Qiagen, Hilden, Germany) and amplified using primers flanking the CRISPR guide sequence within the lentiviral vector (see Supplementary Table S1). PCR products were analyzed on a 1% agarose gel, extracted using the Nucleospin Gel Purification Kit (Macherey & Nagel, Dϋren, Germany) and submitted for Sanger sequencing at the University of Missouri Genomics Technology Core Facility (Columbia, MO). The starting library was additionally subjected to high throughput sequencing as described in section 2.6.

Following qualitative analysis of starting library diversity by Sanger sequencing, the starting library and TZM WT cells were infected with a lethal dose of replication competent HIV-1_NL4-3_ at an MOI of 3, for use of cell death as a partitioning method. When all TZM WT cells had undergone HIV-1_NL4-3_-induced cell death as evidenced by complete detachment from the cell culture dish, remaining viable library cells were washed gently with 1X PBS and allowed to proliferate until the cells reached confluence in a 10 cm dish [Round 1 (R1) population]. R1 cells were collected for genomic DNA isolation as described above and guide sequences were amplified from the integrated vector cassette using primers to append homologous regions (Supplementary Table S1) for InFusion Cloning (Takara Bio USA, San Jose, CA). The PCR amplicons were gel extracted and cloned into the LentiCRISPRv2 vector. Following transformation in STELLAR competent cells, 10 individual bacterial colonies were grown in liquid culture for identification of guide sequences using low throughput Sanger sequencing. The remaining bacterial colonies were pooled, grown in liquid culture, subjected to miniprep, and used as source material for a second round of selection. The resulting R1 library was then used to generate lentiviral particles as described above, followed by transduction of fresh TZM WT cells at a low MOI. After puromycin treatment and proliferation, the cells were again infected with HIV-1_NL4-3_ at an MOI of 3 along with the TZM WT cells as a control. Surviving R1 cells were harvested for genomic DNA isolation and represent the Round 2 (R2) population.

### 2.5 Identification of enriched gRNAs

After completing two rounds of selection, PCR amplicons from the starting library and R2 library were further amplified to append adapters and barcodes for high throughput Illumina sequencing (HTS) at the University of Missouri Genomics Technology Core Facility. HTS was performed on the Illumina HiSeq2000 (Illumina, Inc., San Diego, CA). Data processing was performed by trimming the adaptor regions using cutadapt (Martin, 2011). Importantly, this process discards reads that do not have a gRNA present based on the size of the trimmed sequence. Sequences were then filtered using the FASTXtoolkit^1^ to remove reads with a Phred score at any position below 20. The resulting trimmed and filtered HTS data was then analyzed using FastAptamer-Count (Alam et al., 2015) to count and normalize sequence reads. Sequence reads from the selected libraries were compared to the initial starting library to determine enrichment using FastAptamer-Enrich.

### 2.6 Engineering a TZM-GFP JunB knockout cell line

Lentivirus encoding the JunB-targeted guide sequence (see Supplementary Table S1) was generated by co-transfection of HEK293FT cells using 450 ng LentCRISPRv2-JunB, 450 ng psPAX2, and 100 ng pMD-G complexed with PEI per well in 6-well plates. After 48 h, the virus-containing supernatant was harvested, centrifuged at 1000 × g to remove any cellular debris, transferred to a new tube and frozen at −80°C. The supernatant was then used to transduce fresh TZM WT cells. After 48 h, cells were trypsinized, transferred to a new dish, and cultured with 1 μg/mL puromycin along with TZM WT cells as a control. Puromycin-resistant cells were allowed to proliferate, followed by confirmation of polyclonal modification within JunB. Briefly, genomic DNA was harvested, amplified using JunB-specific primers (Supplementary Table S1), gel extracted, and subjected to Sanger sequencing as previously described. Following confirmation of modification, cells were subjected to single cell isolation to produce clonal isolates. The resulting individual colonies were then expanded for subsequent genomic DNA analysis to identify specific edits as compared to the TZM WT cells.

### 2.7 Western blot

TZM WT and TZM-GFP JunBKO clone 1–6 cells were trypsinized and collected from a 10 cm dish with ice cold PBS. Cells were then centrifuged at 1000 RPM in a 15 mL conical tube. The supernatant was decanted, and any residual supernatant removed by micropipette. To enrich the cellular lysate for nuclear proteins, we performed nuclear fractionation before western blotting. Collected cells were resuspended in 600 μl ice cold TKM Buffer (10 mM Tris–HCl, pH 7.5, 10 mM KCl, 1 mM MgCl2), moved to a pre-chilled microcentrifuge tube, and incubated on ice for 5 min. After incubation, 30 μl of 10% Triton X-100 was mixed into the suspension and incubated for an additional 5 min on ice. The suspension was then centrifuged at 1500 RPM for 5 min at 4° C. The supernatant was gently removed with a micropipette. The pellet was resuspended in 200 μl RIPA buffer (ThermoFisher Scientific, Waltham, MA) containing protease inhibitor (ThermoFisher Scientific, Waltham, MA) and incubated on ice for 20 min before storing overnight at −20°C. A small aliquot of the protein suspension was diluted 1:10 with water to measure protein concentration using the Pierce Coomassie Plus (Bradford) Assay kit (ThermoFisher Scientific, Waltham, MA). 15 μg of protein was then incubated at 95°C for 2 min, separated by SDS-PAGE, and transferred to a PVDF membrane using the Invitrogen Mini Gel Tank System (4–12% NuPage Bis-Tris gel). The PVDF membrane was blocked for 1 h using 5% w/v powdered milk in TBS-T (20 mM Tris, 150 mM NaCl, 0.1% v/v Tween-20, pH 7.6) and probed using a 1:500 dilution of anti-JunB antibody (sc-8051, Santa Cruz Biotechnology, Dallas, TX) in TBS-T containing 5% w/v powdered milk overnight at 4°C. The membrane was then washed three times with fresh TBS-T for 5, 10, and 15 min. The membrane was then incubated at room temperature with anti-mouse HRP antibody (ThermoFisher Scientific, Waltham, MA) at a 1:5000 dilution in TBS-T containing 5% w/v powdered milk for 2 h, and again washed with fresh TBS-T for 5, 10, and 15 min. The membrane was visualized using SuperSignal West Femto Maximum Sensitivity Substrate (ThermoFisher Scientific, Waltham, MA) according to the manufacturer’s instructions. The membrane was then stripped using mild stripping buffer (1.5% w/v glycine, 0.1% w/v SDS, 1% v/v Tween 20, pH 2.2) and re-probed using a 1:1000 dilution of β-actin HRP-conjugated antibody (sc-47778 HRP, Santa Cruz Biotechnology, Dallas, TX) in TBS-T containing 5% w/v powdered milk for 2 h at room temperature. The membrane was visualized as described above.

### 2.8 HIV-1_NL4-3_ infections and viability assays

Viruses were produced as described in section 2.3. To compare infectivity in TZM WT versus JunB KO (polyclonal and clonal) cells, cells were plated in 12-well plates at approximately 40% confluence and infected with indicated dilutions of virus-containing supernatant. Infectivity was determined by measuring the number of GFP-positive cells using flow cytometry at different time points after infection. Briefly, cells were washed gently with 1X PBS and collected from each well using TryplExpress (Gibco, ThermoFisher Scientific, Waltham, MA). Cells were then fixed with 2% paraformaldehyde, washed, and subjected to flow cytometric analysis using a BD Accuri C6 flow cytometer (BD Biosciences, Franklin Lake, NJ). Cell viability assays were performed in 96-well plates 72 h post-infection with serial dilutions of HIV-1_NL4-3_ using the CellTiter96 kit according to the manufacturer’s instructions (Promega, Madison, WI).

### 2.9 Cell surface staining

Indicated cells were collected by removing the culture media from the 10 cm dish, washing the cells with 5 mL 1X PBS and incubating the cells for 10 min in 1 mL 1X PBS containing 10 mM EDTA. Cells were then harvested with PBS, centrifuged at 1000 × *g*, and washed twice with 1 mL 1X PBS. Cells were divided into equal fractions, blocked with 5 mg/mL BSA in 1X PBS for 1 h, and stained with 5 μg primary conjugated antibody according to the manufacturer’s instructions. Antibodies included phycoerythrin (PE)-conjugated CXCR4 (FAB173P, R&D Systems, Minneapolis, MN), PE-conjugated CD4 (RPA-T4, BD Biosciences, Franklin Lakes, NJ), and PE-conjugated IgG2kb isotype control antibody (eBMG2b, Invitrogen, Carlsbad, CA). After staining for 2 h, cells were washed twice with 1 mL 1X PBS and fixed in 2% paraformaldehyde in 1X PBS for 15 min. Cells were again washed twice in 1 mL 1X PBS and analyzed with a BD Accuri C6 Flow Cytometer (BD Biosciences, Franklin Lake, NJ).

### 2.10 Reverse transcription quantitative PCR

Total RNA was collected from TZM WT and TZM-GFP JunB KO clone 1–6 cells using TRIzol (Invitrogen, Carlsbad, CA) according to the manufacturer’s instructions. Isolated RNA was subjected to DNase treatment (Turbo DNase, Ambion) to remove any contaminating genomic DNA and quantified using a NanoDrop Spectrophotometer (ThermoFisher Scientific, Waltham, MA). cDNA was synthesized adding 2 μg cellular RNA to the MutliScribe Reverse Transcriptase kit (ThermoFisher Scientific, Waltham, MA) according to the manufacturer’s instructions. The resulting cDNA samples were then diluted 1:4 in nuclease free water. TaqMan Gene Expression Assays (ThermoFisher Scientific, Waltham, MA) were performed for the detection and quantification of CXCR4, CCR5, and GAPDH in triplicate for three biological samples. Each reaction received: 10 μl PrimeTime qPCR 2X Master Mix (Integrated DNA Technologies, Coralville, IA), 1 μl TaqMan primer/probe, 8 μl nuclease free water, 1 μl diluted cDNA. qPCR was performed using the standard absolute quantification protocol on an Applied Biosystems 7900HT Fast Real-Time PCR System (Applied Biosystems, ThermoFisher Scientific, Waltham, MA).

### 2.11 Single-cycle infectivity assays

Viruses were produced as described above for transduction of TZM WT and TZM-GFP JunB KO clone 1–6 cells. For a subset of experiments, TZM-GFP JunB KO clone 1–6 cells were first plated in a 6-well plate and transfected with 1 μg pcDNA3.1-CXCR4 complexed with PEI. After 48 h, the indicated cells were plated at 250,000 cells per well in a 12-well plate. Cells were then transduced with VSV-G pseduotyped virus, R5-tropic pseudotyped virus or X4-tropic pseudotyped virus. After 48 h, cells were collected, fixed in 2% paraformaldehyde, washed with PBS, and analyzed by flow cytometry on either a BD Accuri C6 flow cytometer or an Attune NxT flow cytometer to determine the percentage of GFP-positive cells.

### 2.12 Chromatin immunoprecipitation

Chromatin immunoprecipitation (ChIP) was performed using the SimpleChIP Enzymatic Chromatin IP Kit (Cat. No. 9003S Cell Signaling Technology, Danvers, MA) according to the manufacturer’s instructions. Briefly, 20 × 10^6^ cells per cell type were crosslinked by addition of 1 mL 10% paraformaldehyde directly into the cell culture dish and quenching with glycine. Cells were collected, washed, and resuspended in Buffer A and incubated on ice with gentle rocking. Nuclei were pelleted by centrifugation at 2,000 × g for 5 min. The pellet was washed with Buffer B and centrifuged again. The pellet was resuspended in Buffer B with Micrococcal Nuclease added and incubated at 37°C for 25 min. The chromatin digestion was stopped by adding 20 μl 0.5 M EDTA. Nuclei were again pelleted by centrifugation. The pellet was resuspended in 1X ChIP buffer. Nuclear membranes were disrupted by sonication in 5 sets of 30 s pulses, with incubation on ice for 2 min between pulses. Nuclear lysates were then centrifuged at 9,400 × *g* for 10 min. A 25 μl sample of the chromatin sample was removed for DNA concentration determination. Into a fresh tube, 1X ChIP buffer was added along with 10 μg of crosslinked chromatin. Ten μl of the indicated antibodies was added to individual tubes (JunB C37F9, YY1 D5D9Z, Histone H3 D2B12, Normal Rabbit IgG #2729, Cell Signaling Technology, Danvers, MA) and rotated overnight at 4°C. To each ChIP reaction, 30 μl protein G magnetic beads were added and rotated for 2.5 h at 4°C. Using a magnetic separation rack, the beads were washed three times with low salt wash buffer and once with high salt wash buffer. Beads were then resuspended in 1X ChIP elution buffer and incubated at 65°C for 45 min with shaking (1,200 RPM). Using a magnetic separation rack, the supernatant was removed and placed in a fresh tube. To the eluate, 6 μl 5 M NaCl and 2 μl proteinase K were added and incubated at 65°C for 2 h, reversing the crosslinks. The DNA was then purified and subjected to end-point PCR using primers specific to the promoter regions for CXCR4, CD4, or CCR5. Primers used for the amplification of the promoter regions are provided in Supplementary Table S2.

## 3 Results

### 3.1 Genome-wide CRISPR screen for HIV-1_NL4-3_ host factors required for infection

**Figure 1:**
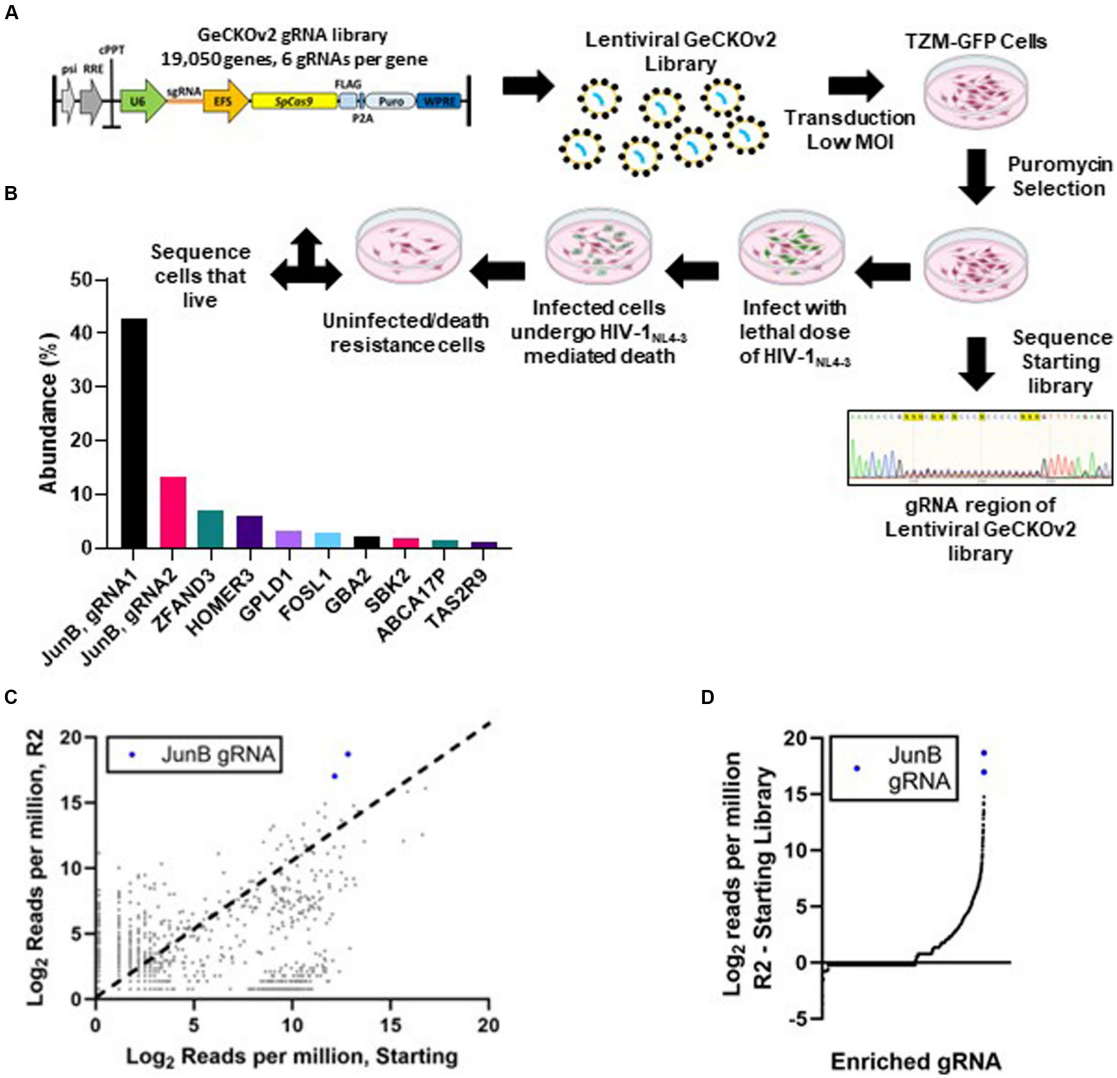
Genome-wide CRISPR/Cas9 screen. **(A)** Schematic of LentiCRISPRv2 HIV host factor screen. Cells were transduced with the LentiCRISPRv2 library (Addgene) at an MOI of 0.1. Transduced cells that survived puromycin selection and continued to proliferate were subjected to a lethal dose of HIV-1_NL4-3_. Surviving cells were subjected to DNA isolation, amplification of the gRNA cassette, and regeneration of the LentiCRISPRv2 library (R1). After two rounds of selection with a lethal dose of HIV-1_NL4-3_ (MOI ~3), the surviving cell population (R2) was subjected to DNA isolation, PCR amplification of the gRNA cassette with Illumina adapters, and high throughput sequencing to identify edited genes. **(B)** Percentage of the total reads of individual gRNAs from HTS sequencing conducted on cells that survived two rounds of selection with a lethal dose of HIV-1_NL4-3_. **(C)** Comparison of normalized reads in both the R2 and starting populations by HTS. Each dot represents a single gRNA. Dots above the dotted line (x = y) were enriched in the selected population, while those below were depleted. **(D)** The difference in normalized reads from the starting library to the R2 selected library for individual genes.

To identify host factors required for HIV-1_NL4-3_ infection, we first created a library of knockout cells (Figure 1A). Wildtype TZM-GFP (TZM WT) cells were utilized because they provide the ability to monitor HIV-1 infection using fluorescence microscopy via HIV long terminal repeat-driven GFP expression (Rosa et al., 2015). To generate the library, TZM WT cells were transduced with lentivirus encoding the human GeCKOv2 library (Shalem et al., 2014) at a low MOI, determined qualitatively per section 2.3, to avoid multiple transductions per cell. While the MOI was estimated to be 0.1, it is possible that multiple transductions were present, despite the relatively low MOI. The GeCKOv2 library contains 19,050 gene targets with 6 gRNAs per gene and the vector encodes a puromycin resistance gene for selection. Following transduction, cells with successful integration events were selected using puromycin. Cells that survived puromycin treatment were allowed to recover and proliferate, representing the starting CRISPR/Cas9 library. As shown in Figure 1A, no single guide RNA was obviously overrepresented in the starting library, with no apparent sequence consensus within the gRNA region of the integrated CRISPR/Cas9 cassette. Rather, the signal intensity given for each individual nucleotide is equivalent across the 20-nucleotide guide RNA region.

Following library generation, we infected TZM WT cells and the starting library with replication competent HIV-1_NL4-3_ at an MOI of 3. We hypothesized that cells with knockout of host factors required for HIV-1_NL4-3_ infection would not be GFP-positive and would therefore survive the lethal dose of HIV-1_NL4-3_. Infection was monitored in the TZM WT cells throughout the selection using fluorescence microscopy to detect HIV-induced GFP expression. When all TZM WT control cells had undergone HIV-induced death and detached from the plate, the surviving cells within the starting library population were allowed to recover and proliferate, representing selection round 1 (R1). Interestingly, we noted that while some of the R1 cells remained presumably uninfected, as they lacked GFP expression, a large majority of the cells were GFP-positive but did not undergo cell death. When the cells reached confluence in a 10 cm dish, genomic DNA was isolated, and the integrated gRNAs were amplified and cloned back into the LentiCRISPRv2 construct for a second round of selection (R2). A subset of the R1 clones were subjected to low throughput sequencing to identify the corresponding genes (Supplementary Table S2) and only one duplicate hit was identified, suggesting that the population maintained some level of diversity and had not completely converged. To further enrich the pool, the remaining R1 bacterial colonies were pooled, subjected to plasmid isolation via miniprep, and used for R2 without adjustments based on gRNA enrichment. Following R2, we again noted that many of the cells were GFP-positive but did not undergo cell death. CRISPR/Cas9 cassettes for the starting library population and the R2 population were subjected to HTS analysis to identify modified genes in the surviving cells.

The resulting HTS data was analyzed using the FastAptamer tool kit (Alam et al., 2015) (see Methods and Materials; Supplementary File S1). JunB represented two of the most abundant gRNAs present in the R2 library with over 650,000 of the 1,160,640 total reads (Figure 1B). Notably, JunB was also identified in the low throughput sequencing performed for the R1 population (Supplementary Table S2). The FastAptamer enrich function compares the two populations by dividing the normalized read count of each gRNA in the starting library by the normalized read count of the same gRNA in the R2 library, yielding the fold change of each gRNA from the starting library to the R2 library. JunB gRNA 1 was enriched 58.2-fold from the starting population to the R2 population, while JunB gRNA 2 was enriched 29.2-fold (Figure 1C). When we compared the difference in normalized reads from the R2 library to the starting library, the two JunB gRNAs had the largest difference in reads out of all the gRNAs (Figure 1D). Together, these data identified JunB as the top candidate for further investigation.

### 3.2 Generation of a TZM-GFP JunB knockout cell line

**Figure 2:**
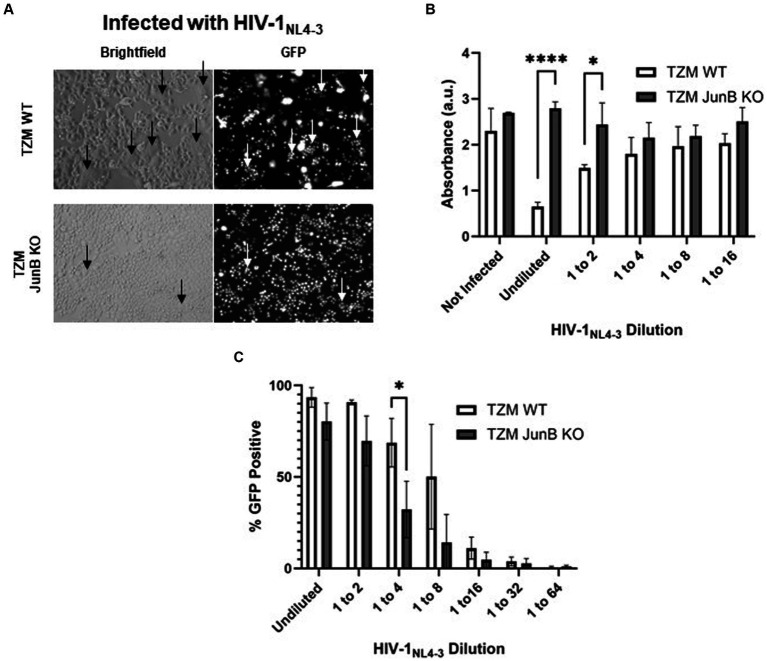
Polyclonal JunB KO cells remained susceptible to HIV-1_NL4-3_ infection but resistant to HIV-induced cell death. **(A)** Representative brightfield and GFP fluorescence microscopy images of TZM WT cells and JunB KO polyclonal cells infected with a lethal dose of HIV-1_NL4-3_ (48-h timepoint). GFP-positive cells are presumed to be infected with HIV-1_NL4-3_. Arrows indicate the presence of syncytia. **(B)** TZM WT cells and JunB KO polyclonal cells were plated in 96-well plates and infected with a lethal dose of HIV-1_NL4-3_ (MOI ~ 3). After 72 h, viability was determined using the CellTiter96 kit. **(C)** Flow cytometry analysis of TZM WT and TZM-GFP JunB KO polyclonal cells infected with dilutions of HIV-1_NL4-3_ in 12-well plates. Error bars represent standard deviation of biological replicates, *n* = 3 (Student’s *t*-test, ****p < 0.0001, *p < 0.05).

We next sought to determine the importance of JunB in HIV-1_NL4-3_ infection. We engineered a polyclonal TZM-GFP JunB knockout (KO) cell line using the JunB gRNA identified in our screen. After confirmation of CRISPR/Cas9 editing, the polyclonal population of TZM-GFP JunB KO cells was used to determine susceptibility to HIV-1_NL4-3_ infection. Like our prior observations in the R1 and R2 libraries, the polyclonal cells were permissive to HIV-1_NL4-3_ infection (Figure 2A) and remained viable after infection with a lethal dose of HIV-1_NL4-3_, in contrast to the TZM WT cells (Figure 2B). Interestingly, at lower input virus amounts, we observed a decrease in GFP-positive cells in the JunB KO cells (Figure 2C), suggesting that JunB KO may impact the ability of the virus to productively infect the cells. Additionally, we observed a reduction in the number of syncytia present within the infected TZM-GFP JunB KO cell population (Figure 2A). Syncytia formation is a hallmark of X4-tropic HIV-1 infection that results from an interaction between the viral envelope glycoprotein expressed on the cell surface and X4 receptors of neighboring cells and contributes substantially to HIV-mediated cell death (Sodroski et al., 1986).

The polyclonal population of TZM-GFP JunB KO cells likely contained numerous heterozygous modifications, including those with the unmodified wildtype sequence, which can present a challenge in evaluating the impact of the knockout. Thus, we generated a clonal TZM-GFP JunB KO cell line to ensure modification of both alleles of the JunB gene (Supplementary Figure S1A). We chose a single clone for further investigation, TZM-GFP JunB KO 1–6, which contained a homozygous addition of cytosine at the −2 position from the protospacer adjacent motif (Supplementary Figure S1B). This edit caused a frameshift mutation in JunB, leading to a missense amino acid sequence. Upon analysis via western blot using an antibody specific for an epitope upstream of the CRISPR/Cas9 target site, we observed that JunB was not detectable in the TZM-GFP JunB KO 1–6 cell line (Supplementary Figures S1C,D).

Of particular importance, we attempted to generate a JunB KO cell line in the CEM-T4 cell line to better model the impact of JunB KO on HIV-1 infection in a more relevant T cell line. CEM-T4 cells were transduced with the lentiviral vector carrying the JunB gRNA in parallel with HEK293FT cells as a control cell population. While we were able to select both polyclonal and clonal HEK293FT JunB KO cells, all CEM-T4 cells died upon selection of transduced cells with puromycin, while a majority of the infected HEK293FT cells remained viable. These results were not due to an inability of CEM-T4 cells to be transduced with VSV-G-pseudotyped lentivirus (data not shown). Thus, we were unable to successfully generate a JunB KO in CEM-T4 cells. We also attempted to perform siRNA knockdown of JunB in the Jurkat and CEM-T4 T cell lines but were unable to achieve knockdown. Collectively, these negative findings support prior studies that have highlighted the importance of JunB in T cells (Koizumi et al., 2018; Hsieh et al., 2021, 2022).

### 3.3 TZM-GFP JunB KO 1–6 resists infection by X4-tropic HIV and does not form syncytia

We next sought to determine the effect of the clonal JunB knockout on susceptibility of the modified cells to HIV-1_NL4-3_ infection. We infected multiple clonal isolates of the JunB KO cell line with HIV-1_NL4-3_. In contrast to the polyclonal population, where we observed that at high MOI, most cells were GFP-positive (Figures 2A,C) but did not succumb to HIV-induced death (Figure 2B), we observed a clear infectivity decrease in the JunB KO cell lines compared to the TZM WT cells (Supplementary Figure S2). As mentioned previously, a decrease in infectivity was also observed in the polyclonal JunB KO cells, albeit at lower input virus amounts (Figure 2C). Of note, while the titer of the virus used in Supplementary Figure S3 was much lower than the virus used in Figure 2, the clonal isolate, JunB KO 1–6 (Supplementary Figure S3), demonstrates greater resistance to infection than the polyclonal population (Figure 2C) when compared wildtype at comparable infectivity levels (~8.5-fold versus ~2.5-fold, respectively). This was unsurprising, as the polyclonal population likely contained unmodified alleles, whereas the clonal JunB KO 1–6 cells contained homozygous edits and were confirmed to lack JunB protein expression. While all subsequent experiments were performed using the TZM-GFP JunB KO 1–6 clonal cells, it is noteworthy that other clonal cells demonstrated similar phenotypes (Supplementary Figure S2). Importantly, the JunB KO 1–6 cells displayed a clear and significant infectivity decrease as compared to TZM WT cells (Supplementary Figure S3).

**Figure 3:**
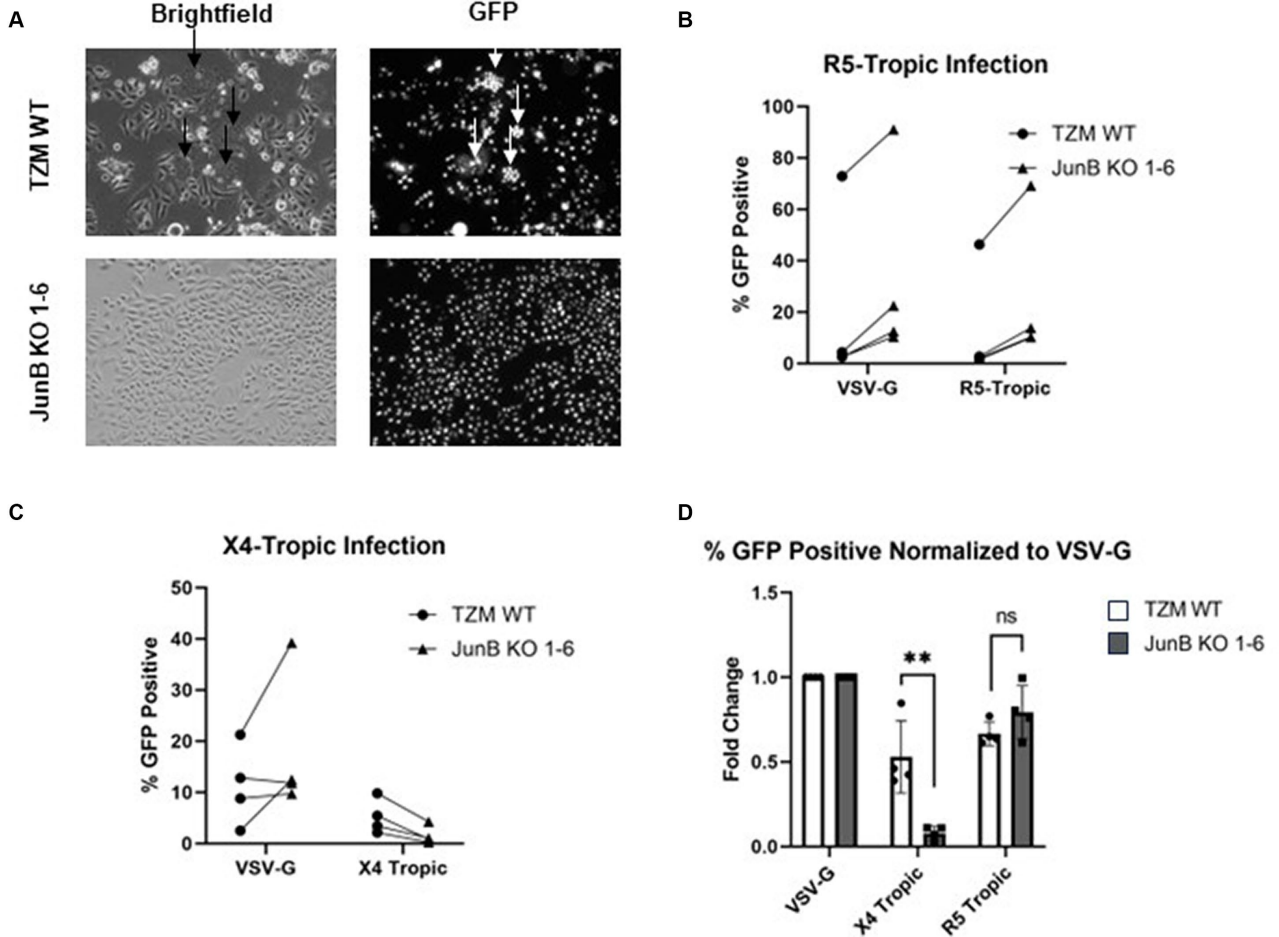
TZM-GFP JunB KO 1–6 cells are resistant to infection of X4-tropic virus. **(A)** Representative brightfield and GFP fluorescence microscopy images of TZM WT and TZM-GFP JunB 1–6 KO cells infected with Env-encoding HIV-1 pseudotyped with VSV-G. Arrows indicate syncytia formation. **(B)** Flow cytometry analysis on TZM WT or TZM-GFP JunB KO 1–6 cells infected with single cycle HIV-1 pseudotyped with VSV-G or R5-tropic Bal envelope. Lines indicate paired replicates where the same virus prep was used to infect the two cell lines. **(C)** Flow cytometry analysis on TZM WT or TZM-GFP JunB KO 1–6 cells infected with single cycle HIV-1 pseudotyped with VSV-G or X4-tropic NL4-3 envelope. Lines indicate paired replicates where the same virus prep was used to infect the two cell lines. **(D)** Normalized flow cytometry analysis on TZM WT or TZM-GFP JunB KO 1–6 cells infected with single cycle HIV-1 pseudotyped with VSV-G, X4-tropic NL4-3 envelope, and R5-tropic Bal envelope. Represented as percent infected cells normalized to percent infected in VSV-G infection. Error bars represent standard deviation of biological replicates (Student’s *t*-test, **p < 0.01).

Because we saw a defect in syncytia formation in the polyclonal population, we next examined syncytia formation in the JunB KO 1–6 cells. Syncytia formation is driven by interactions of surface expressed HIV-1 envelope glycoprotein with CXCR4 and contributes substantially to cell death observed in HIV infection; thus, a decrease in syncytia formation in the JunB KO 1–6 cells would implicate a role for CXCR4 in our observed phenotype. To test syncytia formation in the JunB KO 1–6 cells, we first engineered a lentiviral vector encoding the X4-tropic HIV-1_NL4-3_ envelope, pNL4-3.gag.env. We produced VSV-G pseudotyped viral particles by co-transfecting pNL4-3.gag.env with psPAX2 and pMD-G, and transduced TZM WT and JunB KO 1–6 cells. After 48 h, when the expression of the X4-tropic envelope in the transduced cells was high, we analyzed the cells for syncytia formation using fluorescence microscopy. While it is possible in this experiment that some of the produced viruses carried the X4-tropic envelope encoded by the lentiviral vector, we observed many GFP-positive cells in the JunB KO 1–6 cells (Figure 3A), suggesting that many viruses incorporated VSV-G. We observed a clear defect in the ability of JunB KO 1–6 cells to form syncytia in comparison to the TZM WT cells, which produced many syncytia (arrows, Figure 3A).

As we observed a decrease in syncytia formation in both the polyclonal (Figure 2A) and clonal (Figure 3A) cell lines, we suspected that CXCR4 expression was responsible for the significantly decreased infection observed in the JunB KO 1–6 cells. To further explore this hypothesis, we evaluated the ability of viruses with different tropisms to infect the JunB KO 1–6 cells. We utilized a pseudotyping system to limit variation in viral components other than the envelope glycoprotein. We generated single-cycle viral particles pseudotyped with VSV-G, R5-tropic envelope (Figure 3B), or X4-tropic envelope (Figure 3C) and used them to infect WT and JunB KO 1–6 cells. We then measured the level of infection by quantifying the number of GFP-positive cells using flow cytometry. We hypothesized that if CXCR4 was responsible for the phenotype, VSV-G and R5-tropic viruses would retain their ability to infect the JunB KO 1–6 cells, while the X4-tropic virus would not. Indeed, VSV-G and R5-tropic viruses were able to infect JunB KO 1–6 cells, while X4-tropic cells demonstrated decreased infectivity (Figures 3B,C). To better compare the differences in infection levels across biological replicates, we normalized the percentage of GFP-positive cells in each infection to the percentage of GFP-positive cells in the VSV-G control infection from the same experiment. In this analysis, there was a statistically significant decrease across biological samples in X4-tropic infection between JunB KO 1–6 cells and TZM WT cells, but no difference in R5-tropic infection (Figure 3D). Interestingly, we observed increased levels of GFP-positive cells for both the R5- and VSV-G-pseudotyped viruses in the JunB KO 1–6 cells as compared to TZM WT cells (Supplementary Figure S6). Collectively, these data suggested that X4-tropic HIV was unable to efficiently infect JunB KO 1–6 cells, further implicating CXCR4.

### 3.4 JunB KO 1–6 cells have decreased CXCR4 expression

**Figure 4:**
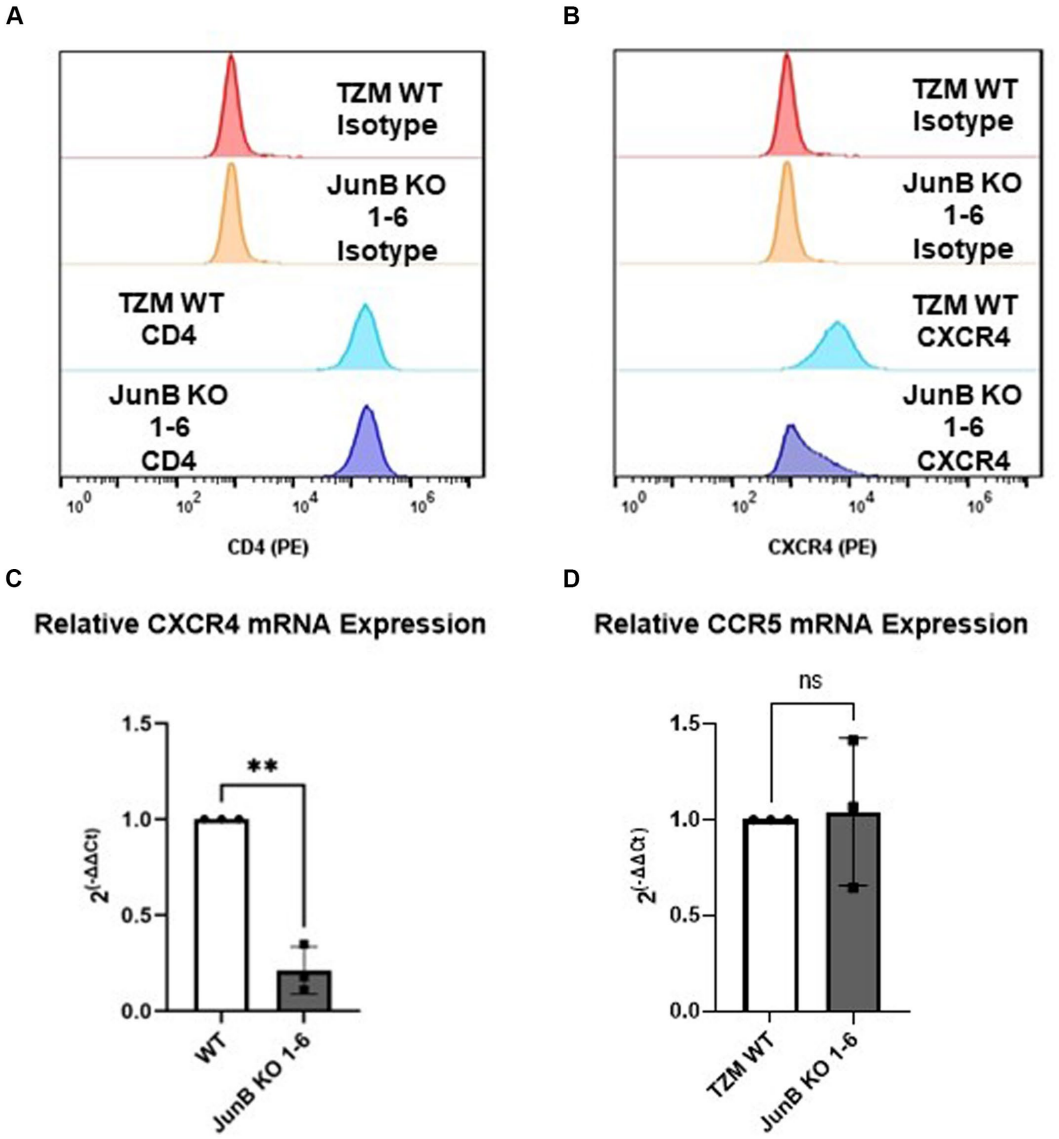
TZM-GFP JunB KO cells have decreased CXCR4 mRNA and protein expression. Flow cytometry analysis of cell surface expression of HIV receptor **(A)** CD4, and **(B)** co-receptor CXCR4 using PE-labeled antibodies. qPCR analyses on mRNA isolated from TZM WT and TZM-GFP JunB KO 1–6 cells for **(C)** CXCR4 mRNA and **(D)** CCR5 mRNA normalized to TZM WT GAPDH mRNA. Each point in **(C,D)** are biological replicates, error bars represent standard deviation. Statistical analysis conducted on non-normalized dCT value as shown in Supplementary Figure S4 (Wilcoxon matched-pairs signed rank test, **p < 0.01). Experiments were repeated three times.

As JunB is a transcription factor, we hypothesized that the absence of JunB may alter the expression of host factors required for viral infection, such as CD4 or CXCR4. To evaluate this possibility, we first examined the expression of CD4 and CXCR4 by surface staining with PE conjugated α-CD4 or α-CXCR4 antibody. We observed no difference in surface expression of CD4 between WT and JunB KO1-6 cells (Figure 4A). This was not entirely surprising, as CD4 is artificially overexpressed in these cells. However, we observed a substantial decrease in α-CXCR4 antibody staining in JunB KO 1–6 cells compared to the WT cells (Figure 4B). We also examined whether knockout of JunB altered CXCR4 mRNA levels. We isolated total RNA from WT and JunB KO 1–6 cells and performed Taqman RT-qPCR analysis to examine the mRNA expression of CXCR4, CCR5, and a housekeeping gene, GAPDH. As expected, the expression of CXCR4 mRNA was significantly decreased in JunB KO 1–6 cells compared to the WT cells (Figure 4C). In contrast, the expression of CCR5 remained unchanged (Figure 4D), supporting our prior observation that R5-tropic viruses retained their ability to infect JunB KO 1–6 cells. These data suggested that the inability of X4-tropic HIV to infect JunB KO 1–6 cells and the lack of syncytia formation in these cells resulted from a decrease in CXCR4 expression at the level of transcription.

### 3.5 Restoration of CXCR4 expression in JunB KO 1–6 cells restores X4-tropic HIV infectivity

**Figure 5:**
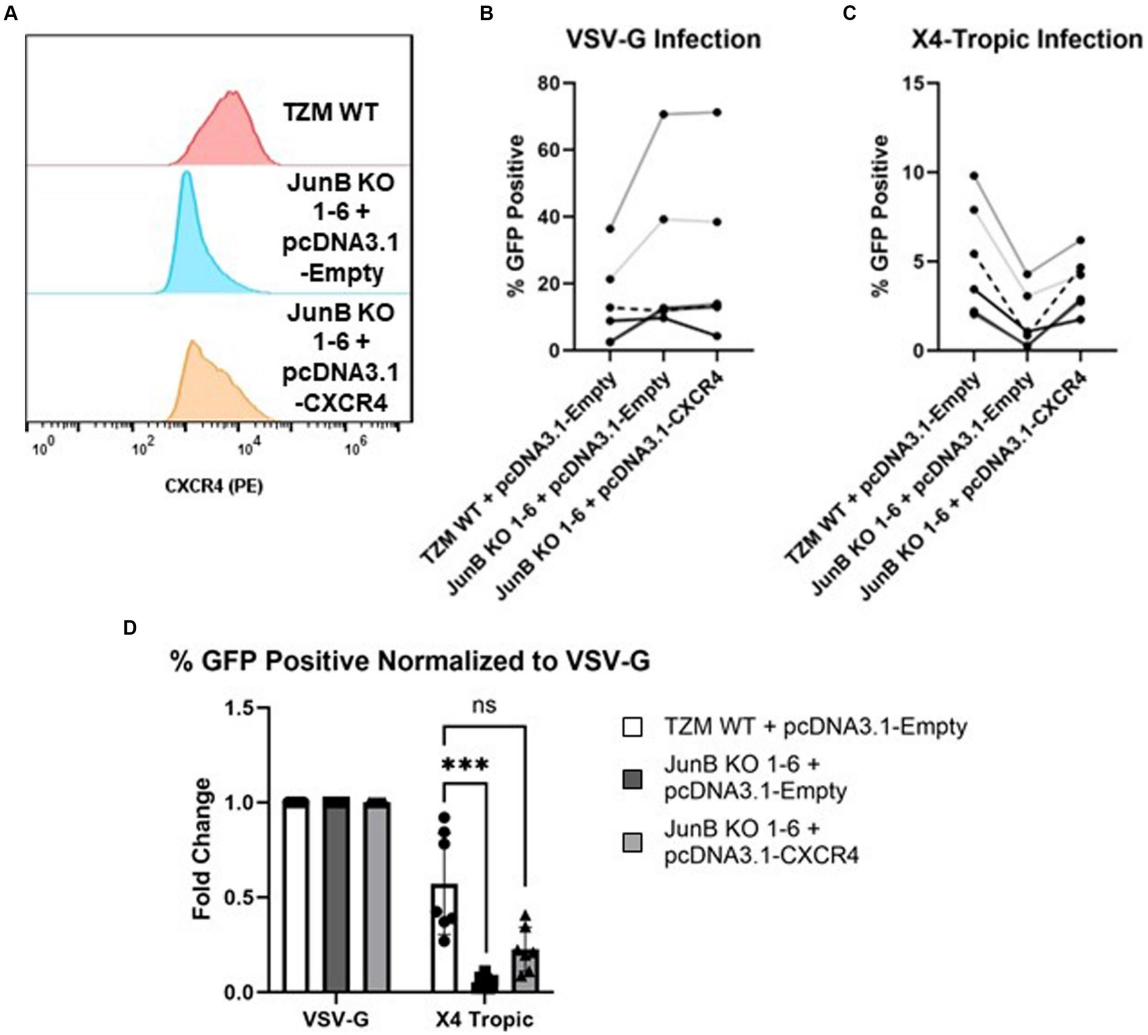
Transfection of exogenous CXCR4 restored the infectivity of X4-tropic HIV. **(A)** Transfection of plasmid pcDNA3.1-CXCR4 restored the surface expression of CXCR4 by flow cytometry using PE-conjugated antibody. A representative experiment of three total experiments is shown. **(B)** Non-normalized flow cytometry analysis of TZM WT or TZM-GFP JunB KO 1–6 cells transfected with either control plasmid pcDNA3.1-empty or plasmid pcDNA3.1-CXCR4 and infected with single cycle HIV-1 pseudotyped with VSV-G. Lines indicate paired replicates where the same virus prep was used to infect the indicated cells. **(C)** Non-normalized flow cytometry analysis of TZM WT or TZM-GFP JunB KO 1–6 cells transfected with either control plasmid pcDNA3.1-empty or plasmid pcDNA3.1-CXCR4 and infected with single cycle HIV-1 pseudotyped with X4-tropic NL4-3 envelope. **(D)** Flow cytometry analysis of cells transfected with control plasmid pcDNA3.1-empty or plasmid pcDNA3.1-CXCR4 and infected with single cycle HIV-1 with the indicated glycoprotein. Represented as percent infected normalized to percent infected in VSV-G infections. Error bars represent standard deviation of biological replicates, *n* = 7. Kruskal–Wallis test, ***p < 0.001.

To confirm that the decrease in CXCR4 expression was specifically responsible for the lack of X4-tropic viral infection in JunB KO 1–6 cells, we recovered CXCR4 expression by transfection of a CMV-driven CXCR4 expression plasmid. Notably, we did consider attempting to restore JunB expression in these cells. However, given the complexities surrounding the role of JunB in directly or indirectly activating and/or repressing the expression of numerous genes involved in diverse cellular processes, data interpretation could present a significant challenge (Chiu et al., 1989; Shaulian and Karin, 2001; Shaulian and Karin, 2002; Pérez-Benavente et al., 2013; Garces de los Fayos Alonso et al., 2018). Thus, we chose to restore CXCR4 expression, rather than JunB expression, to determine whether CXCR4 expression was a specific contributor to our observed phenotype. Recovery of cell surface expression was determined by surface staining with an α-CXCR4 PE conjugated antibody (Figure 5A and Supplementary Figure S5). Following confirmation of CXCR4 surface expression, we infected cells with single-cycle HIV-1 pseudotyped with either an VSV-G (Figure 5B) or X4-tropic (Figure 5C) envelope and measured infection rates by flow cytometry as previously described. Although in a representative experiment, the transfection of CXCR4 did not restore surface CXCR4 expression to the level of TZM WT (Supplementary Figure S5, 71.4% in TZM WT versus 66.5%), X4-tropic virus was able to infect JunB KO 1–6 cells transfected with the plasmid encoding CXCR4 nearly to that of the TZM WT cells (Figures 5C,D and Supplementary Figure S6A). Collectively across all experiments, transfection of the JunB KO 1–6 cells with a plasmid encoding CXCR4 supported a corresponding increase in the number of GFP-positive cells (Figure 5D), suggesting that the susceptibility of the cells to infection with X4-tropic virus increased with increasing CXCR4 expression. Further, while transfection of a CXCR4-expressing plasmid increased susceptibility of JunB KO 1–6 cells to X4-tropic infection as compared to the empty vector control, it did not alter the susceptibility of these cells to R5-tropic, or VSV-G pseudotyped virus (Supplementary Figure S6). These data further support our conclusion that decreased CXCR4 expression in JunB KO 1–6 is responsible for the observed block to HIV-1_NL4-3_ infection.

### 3.6 Direct versus indirect interactions of JunB with the CXCR4 promoter

**Figure 6:**
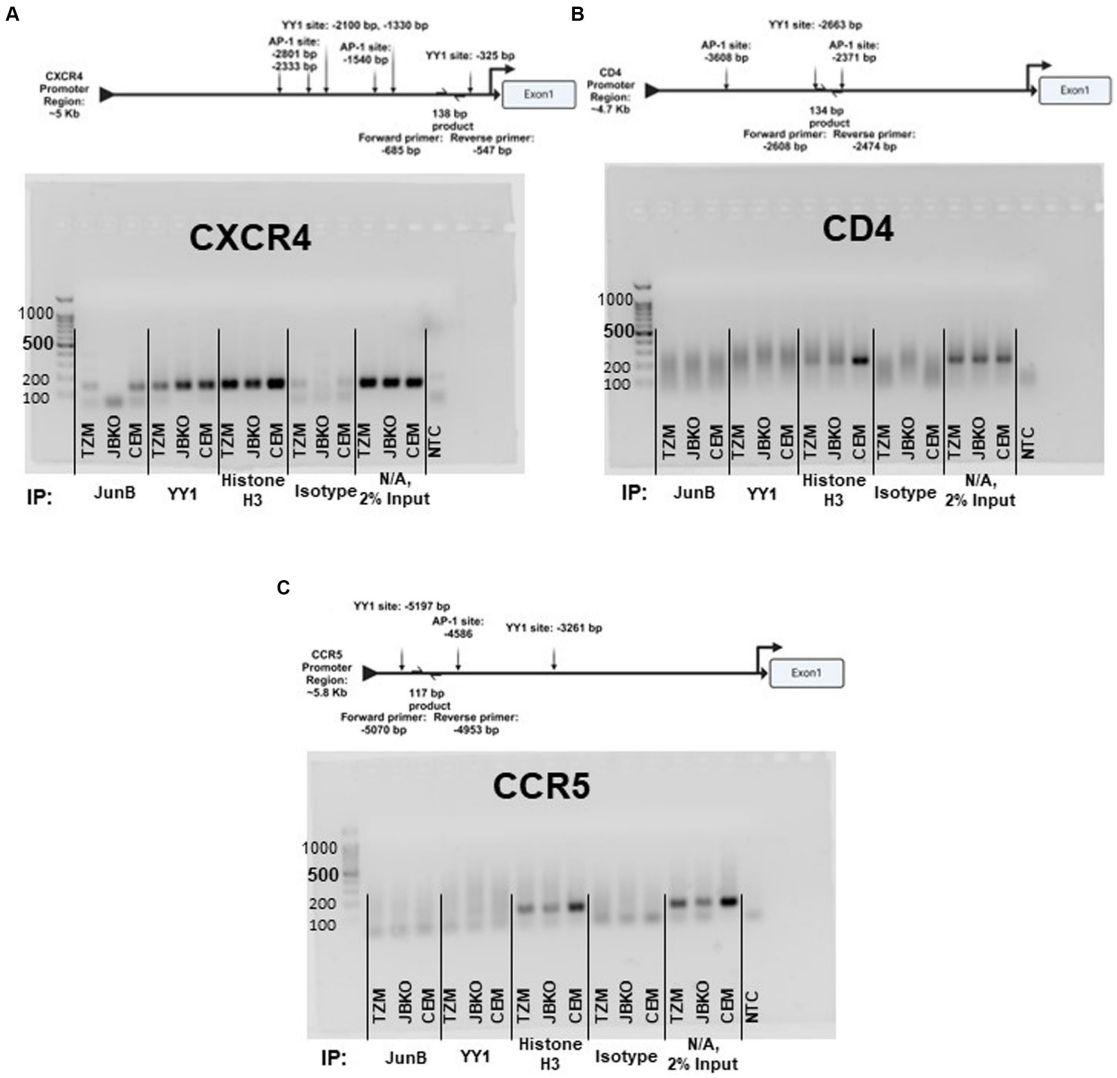
Promoter schematics and representative images of 2% agarose gel electrophoresis of PCR products corresponding to the promoter region of **(A)** CXCR4, **(B)** CD4, and **(C)** CCR5 using DNA recovered from chromatin immunoprecipitation assays in TZM WT, TZM-GFP JunB KO 1–6 (JBKO), and CEM T4 (CEM) cells.

As our data suggested downmodulation of CXCR4 expression by JunB, we sought to determine whether JunB interacts directly with the CXCR4 promoter. We performed ChIP experiments using the TZM WT cells, the JunB KO 1–6 cells, and the CEM-T4 T cell line (Figure 6). While we were unable to study the JunB KO phenotype in Jurkat or CEM-T4 cells as discussed in section 3.2, we reasoned that examination of JunB occupancy of the CXCR4 promoter in these cells might illuminate a potential role for JunB in these more relevant cells. We included the CD4 and CCR5 promoters as controls, as our data suggested they were not subject to JunB modulation in our model. We also included a ChIP using a Histone H3 specific antibody as an assay control. Following amplification, we observed bands of the appropriate size for the CXCR4 promoter region in our TZM WT and CEM-T4 JunB samples, but not in our JunB KO 1–6 sample, suggesting that JunB occupies the CXCR4 promoter in TZM WT and CEM-T4 cells (Figure 6A). However, we also observed some background signal in our isotype control samples. Of note, we did not observe amplification for CD4 (Figure 6B) or CCR5 (Figure 6C) in any of the cell lines for our JunB sample set. In addition to direct interaction, we explored potential indirect modulation through examination of promoter occupancy by Ying Yang 1 (YY1), which has been reported to be a repressor of CXCR4 (Hasegawa et al., 2001; Tarnowski et al., 2010). YY1 can be upregulated by c-Jun (Balkhi et al., 2018), and it has been reported that c-Jun can be negatively regulated by JunB (Chiu et al., 1989; Schütte et al., 1989; Deng and Karin, 1993). Thus, in the absence of JunB, CXCR4 could be downregulated by the lack of repression of c-Jun, which would potentially increase expression of the CXCR4 repressor, YY1. We observed bands of the appropriate size for each YY1 sample, suggesting that YY1 occupies the CXCR4 promoter in all three cell lines (Figure 6A). Interestingly, the band intensity was increased for the JunB KO 1–6 sample versus the TZM WT sample. Collectively, these data suggest there is more to learn about the mechanism by which JunB modulates CXCR4 expression, as evidence exists for both direct and indirect interactions.

## 4 Discussion

In this study, we utilized the CRISPR/Cas9 system to conduct a high throughput screen for host factors required for HIV-1_NL4-3_ infection using cell death as a partitioning method. Cells which contained the gRNA for JunB represented an overwhelming majority of the cells surviving the lethal dose of HIV-1_NL4-3_, suggesting that the presence of JunB was important for HIV-1_NL4-3_ induced death. Upon generation of a clonal JunB KO cell line, we demonstrated that X4-tropic virus had significantly decreased infectivity in the JunB KO 1–6 cell line. Furthermore, the JunB KO 1–6 cells were unable to support the formation of syncytia upon expression of X4-tropic HIV Env, a process that requires CXCR4. This phenomenon can be explained by our observation of decreased CXCR4 transcription and protein expression in JunB KO 1–6 cells and is further supported by the observation that recovery of CXCR4 expression in these cells restored the infectivity of X4-tropic HIV.

The CRISPR/Cas9 system is a powerful tool that is often used to study the function(s) of individual proteins in cells. While our system generates loss-of-function mutations, other CRISPR systems are now capable of incorporating gain-of-function or overexpression. In each of these systems, the impact of the modification in comparison with wildtype controls can be powerfully informative. Using a library of gRNAs provides the ability to screen in a high throughput manner for proteins vital to specific processes when combined with relevant partitioning methods. Here, we sought to identify proteins required for HIV-1_NL4-3_ infection using HIV-1_NL4-3_-mediated cell death as a partition. Loss-of-function of required proteins in this system could result in the inability of HIV-1_NL4-3_ to induce cell death or to productively infect the cell. Conspicuously, the infection could be blocked by a variety of different mechanisms. Our screen overwhelmingly identified a transcription factor, JunB, that both prevented HIV-induced cell death and decreased HIV-1_NL4-3_ infection. Notably, these two phenotypes were linked. Our data suggest that cells lacking JunB display a decreased expression of CXCR4, impacting both the formation of syncytia, which plays a large role in HIV-induced cell death, and the ability of X4-tropic viruses to infect the JunB KO cells. At the polyclonal level, where homozygous and heterozygous edits exist, it was likely that the decrease in CXCR4 expression was enough to reduce syncytia formation, but not enough to completely block infection. As syncytia formation plays a substantial role in HIV-driven cell death, the polyclonal cells were thus seemingly resistant to HIV-induced death. In contrast, the decrease in CXCR4 expression in the clonal JunB KO 1–6 cells was substantial enough to significantly inhibit infection. These results have important implications in the analysis of knockout cell lines.

Our selection method, while unique, was not without experimental biases. Cells were allowed to proliferate after treatment with HIV-1_NL4-3_ to increase the enrichment of the surviving population; however, this also biased the selection toward cells which proliferate faster. Indeed, we observed that the JunB KO cells proliferated at a higher rate than that of TZM WT cells (data not shown). This increase in proliferation rate, along with their infectivity phenotype, likely caused the JunB KO cells to dominate the selected population. The opposite effect could also introduce bias, as edits that resulted in decreased proliferation rates would cause depletion of those cells from the selected population despite a positive infectivity phenotype (i.e., lack of infection). This bias may have resulted in our lack of identification of host factors previously determined to be required for HIV-1_NL4-3_ infection, such as CD4 (Arthos et al., 1989) or CXCR4 (Feng et al., 1996). Notably, CXCR4 expression can drive the proliferation of cells in several different tissues (Bianchi and Mezzapelle, 2020) and the knockdown of CXCR4 can inhibit cell proliferation (Hong et al., 2008). Thus, knockout of CXCR4 could prevent the proliferation of cells within the selected library, which would be quickly overwhelmed by the increased proliferation rate of the JunB KO cells. In the case of CD4, it is notable that the TZM WT cell line is engineered to overexpress CD4. Thus, it is possible that the CRISPR/Cas9 machinery was unable to overcome the high level of CD4 expression in these cells, precluding our identification of CD4 in our screen. Despite the lack of expected observations within our screen, we were able to identify a novel host factor indirectly involved in HIV infection and our data supporting the transcriptional control of CXCR4 by JunB are independent of the selection outcomes.

Interestingly, the JunB KO 1–6 cells appeared to have increased susceptibility to infection with VSV-G-pseudotyped and R5-tropic envelope-pseudotyped viruses (Supplementary Figure S6). Our data support downmodulation of CXCR4 by JunB, but it is likely that other host factors could be affected by knockout of JunB. Further, it is well documented in the literature that AP-1 transcription factor family members, including JunB, play a significant role in regulation of proviral transcription (Mercier et al., 1994; Canonne-Hergaux et al., 1995; Roebuck et al., 1996; Kaczmarek et al., 2013; van der Sluis et al., 2014). Thus, it is possible that the increase in GFP expression detected in JunB KO 1–6 cells may be due to resulting impacts on proviral transcription. Furthermore, the AP-1 transcription factor family has been shown to have direct and dueling impacts on viral latency (Cobos Jiménez et al., 2023). It is possible that the loss of function of JunB in our model could impact reactivation of latent virus, and merits further analysis of the role played by JunB at different stages of HIV infection. The decrease in infection of X4-tropic HIV and apparent increase in susceptibility to R5-tropic infection (Supplementary Figure S6) highlights the potential of AP-1 to have seemingly conflicting effects depending on stage of infection (Cobos Jiménez et al., 2023), which must be carefully explored.

Here, we present data that further clarifies the specific roles of JunB in human cells. The redundant nature of the AP-1 transcription factor family presents several challenges, and the promiscuity of each family member may conceal gene targets. These data highlight a role for the AP-1 transcription factor family in the regulation of CXCR4, but additional studies are needed to fully understand the impact of these transcription factors on CXCR4 expression. Although characterization of the CXCR4 promoter region identified a potential c-Jun binding sequence (Caruz et al., 1998), to our knowledge, there is no prior data directly examining the control of CXCR4 by JunB. However, it has been reported that transcription factor YY1 is a repressor of CXCR4 (Hasegawa et al., 2001; Tarnowski et al., 2010). This is notable, as YY1 can be upregulated by c-Jun (Balkhi et al., 2018) and it has been reported that c-Jun can be negatively regulated by JunB (Chiu et al., 1989; Schütte et al., 1989; Deng and Karin, 1993). Thus, YY1 provides a possible indirect mechanism for JunB downmodulation of CXCR4. With this in mind, we explored CXCR4 promoter occupancy by both JunB and YY1 using ChIP. For JunB, we observed bands of the appropriate size for the TZM WT and CEM-T4 samples, but not for the JunB KO 1–6 sample, suggesting a direct interaction of JunB with the CXCR4 promoter; however, slight background in the isotype controls was also present. Regardless, these data suggest that direct JunB regulation of the CXCR4 promoter in other T cell lines and primary T cells may be worth further study, as bands were clear in the CEM-T4 sample. Interestingly, our results also demonstrated an increase in CXCR4 promoter occupancy for YY1 in JunB KO 1–6 cells as compared to TZM WT or CEM-T4 cells. Collectively, it is possible that both direct and indirect mechanisms are playing a role in downmodulation of CXCR4 by JunB, demonstrated by reduced promoter occupancy of CXCR4 by JunB and additional repression of CXCR4 by YY1 in JunB KO 1–6 cells. We did not observe occupancy of either the CD4 or CCR5 promoters by JunB, despite prior reports that JunB regulates the CCR5 promoter in some cells types (Hasan et al., 2017; Wheaton and Ciofani, 2020). The lack of amplification at the examined CD4 site in the Histone H3 ChIP may indicate that in HeLa cells, this site is inaccessible, mutated, or contains other epigenetic differences that would impact amplification. It is important to note that the ChIP analysis detailed here focused on the canonical AP-1 sequence, TGA(C/G)TCA (Bohmann et al., 1987). However, there are many additional sites present in the promoter regions of CXCR4, CD4, and CCR5 that are within one nucleotide similarity of the AP-1 consensus sequence, which may also permit binding of AP-1 family members (Friling et al., 1992). Thus, additional studies will be required to fully elucidate the mechanism by which JunB modulates CXCR4, CCR5, and CD4 promoters in these different cell types. Interestingly, another member of the AP-1 transcription factor family, FOSL1, was identified within the top 10 gRNAs in our selected population (Figure 1A). This may implicate a role for a JunB/FOSL1 heterodimer in our observed phenotype and provides another direction for future studies.

While we were hopeful our screen could identify potential therapeutic targets for HIV, the JunB-CXCR4 axis does not represent a promising candidate for therapeutic development. JunB is known to affect cell proliferation and survival, a phenotype not always conserved across cell lines, making its perturbation potentially problematic *in vivo*. Specifically, in JunB deficient mice, T cells become more sensitive to T cell receptor mediated apoptosis, which could potentially exacerbate the progression of AIDS in an HIV infected patient (Koizumi et al., 2018; Hsieh et al., 2021). We attempted to replicate our findings in a more HIV-relevant cell line, CEM-T4, but were unable to successfully generate a JunB KO in CEM-T4 cells. We were able to generate a clonal JunB KO in HEK293FT cells in parallel, suggesting that our lentiviral system was viable. Of note, VSV-G-pseudotyped lentivirus is capable of infecting CEM-T4 cells. However, the death of all CEM-T4 cells that initially survived puromycin selection supports prior data demonstrating the importance of JunB in the expansion and survival of T-cell populations (Hsieh et al., 2021, 2022). We further attempted knockdown of JunB expression using siRNA in both Jurkat and CEM-T4 cells. While we did not observe significant cell death in these populations, we were also unable to observe protein knockdown. In this case, it is possible that cells that did have successful knockdown of JunB quickly became apoptotic and were removed from the population before analysis could begin.

The transcriptional control of CXCR4 by JunB observed here was characterized in the HeLa-based TZM-GFP cell line. It is possible that the same phenomenon would not be observed in other cell types. However, we serendipitously characterized a phenomenon that could be impactful outside of the HIV context. Data from cancer studies may support JunB modulation of CXCR4 in other cell types. Both JunB and CXCR4 have been shown to be overexpressed breast cancer (Kallergi et al., 2019, 2020), and CXCR4 has been implicated in tumor progression in pancreatic cancer (Dubrovska et al., 2012). In addition, it has been shown that CXCR4 expression, along with JunB mRNA expression, is increased in a 3D spheroid cell culture model (Kallergi et al., 2020). While this phenomenon has not yet been further explored, it may agree with our data supporting that JunB is involved in the regulation of CXCR4.

## Data availability statement

Illumina sequencing data sets are available on the Open Science Framework data repository: https://osf.io/pazrj/.

## Ethics statement

Ethical approval was not required for the studies on humans in accordance with the local legislation and institutional requirements because only commercially available established cell lines were used.

## Author contributions

WS: Formal analysis, Methodology, Validation, Visualization, Writing – original draft, Writing – review & editing. DG: Formal analysis, Writing – review & editing. MJ: Conceptualization, Funding acquisition, Methodology, Supervision, Writing – review & editing. ML: Conceptualization, Data curation, Formal analysis, Investigation, Methodology, Project administration, Resources, Supervision, Validation, Visualization, Writing – review & editing, Funding acquisition.

## Footnotes

^1^ http://hannonlab.cshl.edu/fastx_toolkit/
