# Supplementary material for: Genome-wide CRISPR/Cas9 screen reveals JunB downmodulation of HIV co-receptor CXCR4
Source: Front Microbiol. 2024 May 20;15:1342444. doi: 10.3389/fmicb.2024.1342444 (PMC11149427; doi:10.3389/fmicb.2024.1342444)
Supplement: Supplementary file 1 [file Data_Sheet_1.pdf]

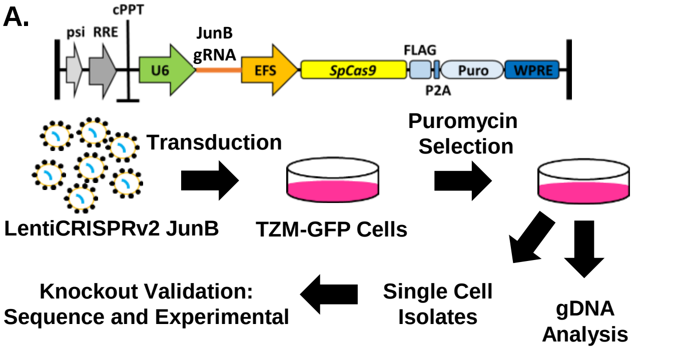

B. Wildtype and modified JunB nucleotide sequence

JunB Guide 2

```
GCATCGCGCGCCTGGAGGACAAGGTGAAGACGCTCAAGGCCGAGAACGCGGGGCTGTCGAGTACC-GCCGGCCTCCTCCGGGAG   Wildtype
|||||||||||||||||||#||||||||||||||||||||||||||||||||||||||||||||||||| ||||||||||||||||||
GCATCGCGCGCCTGGAGGANAAGGTGAAGACGCTCAAGGCCGAGAACGCGGGGCTGTCGAGTACCCGCCGGCCTCCTCCGGGAG   JunB KO 1-6
```

C. Wildtype and modified JunB protein sequence

Wildtype:

MCTKMEQPFYHDDSYTATGYGRAPGGLSLHDYKLLKPSLAVNLADPYRSLKAPGARGPGPEGGGGGSYFSGQGSD
TGASLKLASSELERLIVPNSNGVITTTPTPPGQYFYPRGGGSGGGAGGAGGGVTEEQEGFADGFVKALDDLHKMN
HVTPPNVSLGATGGPPAGPGGVYAGPEPPPVYTNLSSYSPASASSGGAGAAVGTGSSYPTTTISYLPHAPPFAGG
HPAQLGLGRGASTFKEEPQTVPEARSRDATPPVSPINMEDQERIKVERKRLRNRLAATKCRKRKLERIARLEDKV
KTLKAENAGLSST**AGLLREQVAQLKQKVMTHVSNGCQLLLGVKGHAF**

JunB KO 1-6:

MCTKMEQPFYHDDSYTATGYGRAPGGLSLHDYKLLKPSLAVNLADPYRSLKAPGARGPGPEGGGGGSYFSGQGSD
TGASLKLASSELERLIVPNSNGVITTTPTPPGQYFYPRGGGSGGGAGGAGGGVTEEQEGFADGFVKALDDLHKMN
HVTPPNVSLGATGGPPAGPGGVYAGPEPPPVYTNLSSYSPASASSGGAGAAVGTGSSYPTTTISYLPHAPPFAGG
HPAQLGLGRGASTFKEEPQTVPEARSRDATPPVSPINMEDQERIKVERKRLRNRLAATKCRKRKLERIARLEDKV
KTLKAENAGLSST**RRPPPGAGGPAQTEGHDPRQQRLSAAAWGQGTRLLNVPCPFTDTPSLGRLGTRLPLGSREQA**
VGTHPGT

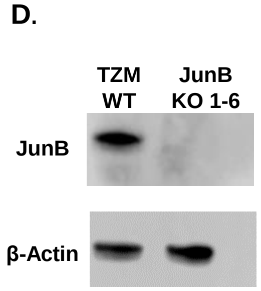

Figure S1: Successful knockout of JunB in TZM-GFP JunB KO 1-6. A. Schematic of production of the TZM-GFP JunB KO cells. LentiCRIPSRv2 containing the JunB gRNA used to transduce TZM-GFP cells. Cells surviving puromycin contain the editing machinery and are isolated by plating sparsely and growing individual colonies. Clones then have gDNA isolated and the JunB target location amplified by PCR. The resulting genomic sequences are then sequenced by Sanger sequencing. B. Identification of a single cytosine insertion in TZM-GFP JunB KO 1-6 cells. C. JunB protein sequences of the TZM-GFP Wildtype and TZM-GFP JunB KO 1-6 cells. D. Western blot of TZM-GFP Wildtype and TZM-GFP JunB KO 1-6 nuclear enriched cell lysates.

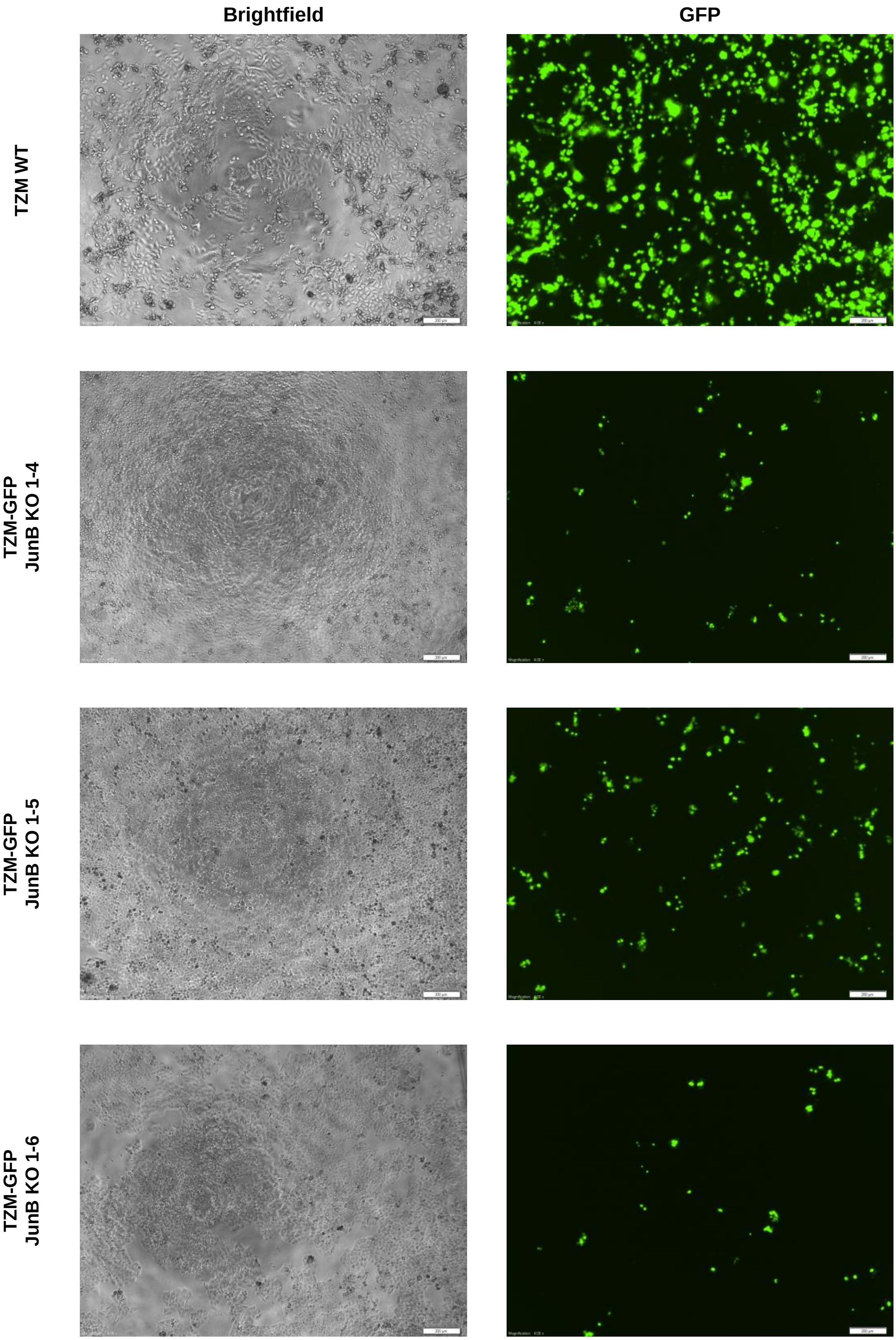

Figure S2: Single cell isolates of TZM-GFP JunB KO cells are resistant to HIV-1$_{NL4-3}$ infection. Representative images of brightfield and fluorescent (GFP) microscopy of TZM-GFP and TZM-GFP JunB KO single cell clones infected with HIV-1$_{NL4-3}$.

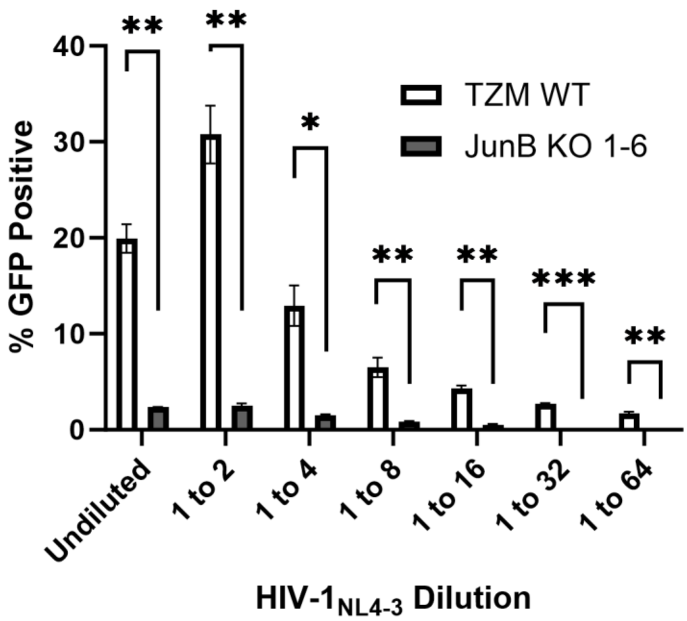

Figure S3: TZM-GFP JunB KO 1-6 is resistant to HIV-1_{NL4-3} infection. Flow cytometry analysis of TZM-GFP cells and a clonal isolate of the TZM-GFP JunB KO, clone 1-6, infected with HIV-1_{NL4-3}. Error bars represent standard deviation of biological replicates, n=3 (Student's T test, *P>0.05, **P>0.01, ***P>0.001).

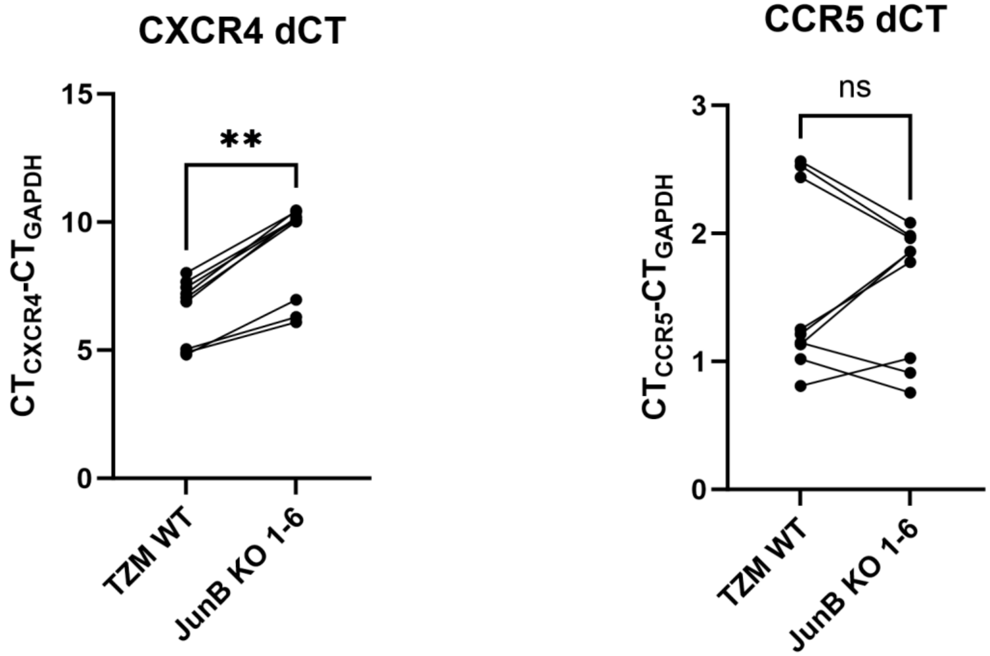

Figure S4: qPCR cycle threshold (CT) values of CXCR4 or CCR5 subtracted from the CT value of GAPDH in both TZM WT and JunB KO 1-6 cells. A lower dCT indicates higher expression of the gene of interest. Experiments conducted in triplicate three separate times. Each point indicates an individual replicate, n=9 (Wilcoxon matched-pairs signed rank test, **P>0.01)

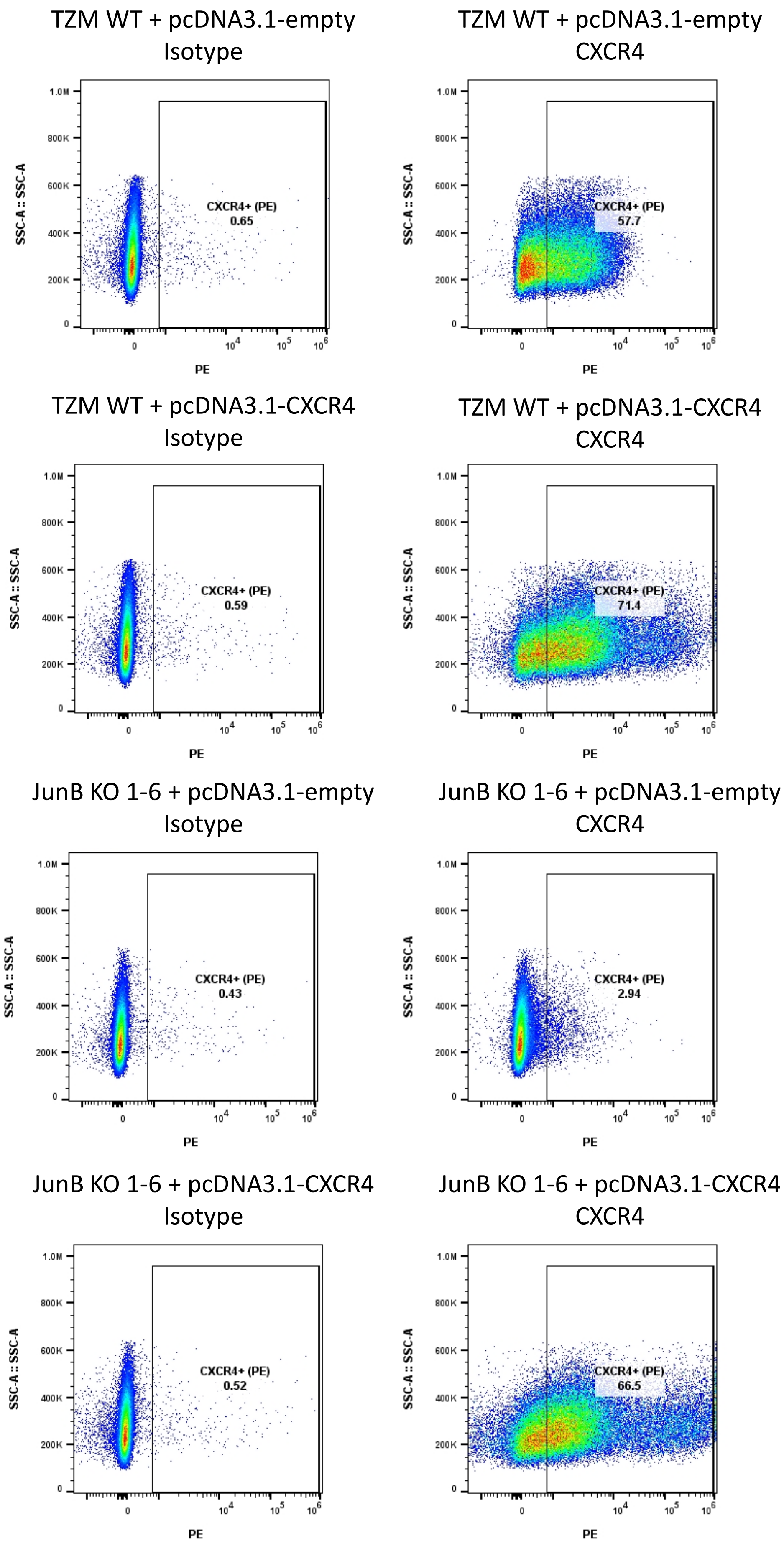

Figure S5: Transfection of pcDNA3.1-CXCR4 recovers surface expression of CXCR4 in JunB KO 1-6 cells. Flow cytometry plots cells for PE staining of surface expressed CXCR4. Cells transfected with the empty pcDNA3.1-empty vector or pcDNA3.1-CXCR4, stained with either an isotype control PE-labeled antibody or an anti-CXCR4 PE-labeled antibody.

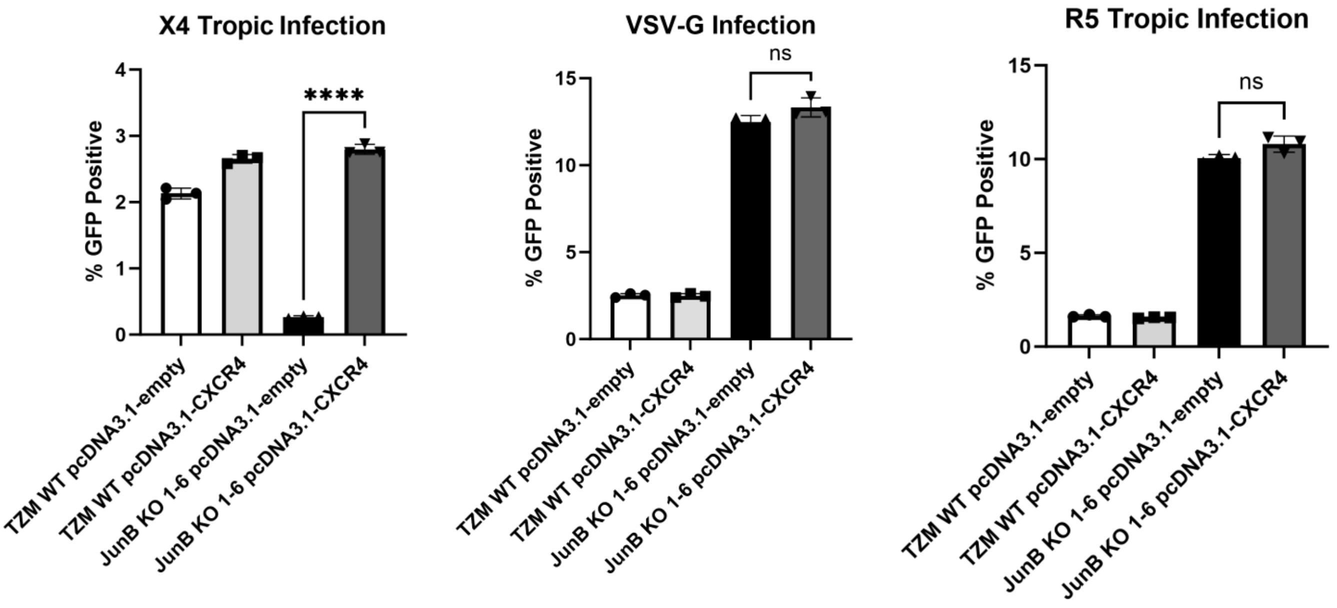

Figure S6: JunB KO 1-6 cells have a greater susceptibility to infection of non-X4 tropisms over wild type. TZM-GFP WT or TZM-GFP JunB KO 1-6 cells were transfected with either the empty vector, pcDNA3.1-empty, or pcDNA3.1-CXCR4 and infected with virus with the indicated tropism. Flow cytometry analysis of GFP expressing cells. Representative data from CXCR4 addback experiments. Error bars represent the standard deviation of technical replicates (Student's t-test, ****$P<0.0001$)
